# Brain Cancer Prediction Based on Novel Interpretable Ensemble Gene Selection Algorithm and Classifier

**DOI:** 10.3390/diagnostics11101936

**Published:** 2021-10-19

**Authors:** Abdulqader M. Almars, Majed Alwateer, Mohammed Qaraad, Souad Amjad, Hanaa Fathi, Ayda K. Kelany, Nazar K. Hussein, Mostafa Elhosseini

**Affiliations:** 1 College of Computer Science and Engineering, Taibah University, Yanbu 46411, Saudi Arabia; Amars@taibahu.edu.sa (A.M.A.); MWATEER@taibahu.edu.sa (M.A.); 2 Department of Computer Science, Faculty of Science, Abdelmalek Essaadi University, Tetouan 93000, Morocco; s.amjad@uae.ac.ma; 3 Math and Computer Science Department, Amran University, Amran 891-6162, Yemen; 4 Math and Computer Science Department, Menoufia University, Menoufia 32511, Egypt; Hanaa_4_ever@yahoo.com; 5 Department of Genomic Medicine, Faculty of Science, Cairo University, Cairo 12613, Egypt; aidakelany@gmail.com; 6 Department of Mathematics, College of Computer Sciences and Mathematics, Tikrit University, Tikrit 34001, Iraq; nazar.dikhil@tu.edu.iq; 7 Computers Engineering and Control Systems Department, Faculty of Engineering, Mansoura University, Mansoura 35516, Egypt

**Keywords:** gene expression data, brain cancer, classification, ensemble methods, hyperparameter optimization, feature selection, gene selection

## Abstract

The growth of abnormal cells in the brain causes human brain tumors. Identifying the type of tumor is crucial for the prognosis and treatment of the patient. Data from cancer microarrays typically include fewer samples with many gene expression levels as features, reflecting the curse of dimensionality and making classifying data from microarrays challenging. In most of the examined studies, cancer classification (Malignant and benign) accuracy was examined without disclosing biological information related to the classification process. A new approach was proposed to bridge the gap between cancer classification and the interpretation of the biological studies of the genes implicated in cancer. This study aims to develop a new hybrid model for cancer classification (by using feature selection mRMRe as a key step to improve the performance of classification methods and a distributed hyperparameter optimization for gradient boosting ensemble methods). To evaluate the proposed method, NB, RF, and SVM classifiers have been chosen. In terms of the AUC, sensitivity, and specificity, the optimized CatBoost classifier performed better than the optimized XGBoost in cross-validation 5, 6, 8, and 10. With an accuracy of 0.91±0.12, the optimized CatBoost classifier is more accurate than the CatBoost classifier without optimization, which is 0.81± 0.24. By using hybrid algorithms, SVM, RF, and NB automatically become more accurate. Furthermore, in terms of accuracy, SVM and RF (0.97±0.08) achieve equivalent and higher classification accuracy than NB (0.91±0.12). The findings of relevant biomedical studies confirm the findings of the selected genes.

## 1. Introduction

Brain cancer is the leading cause of death in women under the age of 20 and men under 40 [1,2]. Moreover, the prevalence of malignant brain tumors is rising [3], severely impacting society and human health [4]. Primary brain tumors arise from the brain cells themselves, while secondary brain tumors arise from malignant cells outside of the brain and spread there [5]. According to studies, brain tumors are extremely heterogeneous, posing a major challenge for classification, segmentation, diagnosis, and prognosis [6]. 

Microarray-based gene expression profiling has proven useful for cancer detection, prognosis, and treatment [7]. In addition, in recent years, DNA microarray technology has significantly impacted the information that we have bout what causes cancer [8,9]. 

Cancer microarray data typically contain a small number of samples with many gene expression levels as features, which leads to the curse of dimensionality, making the classification of microarray data a difficult task. The bioinformatics community uses a variety of approaches to classify microarray data using machine learning systems. Most of the examined studies on cancer classification using microarray data sets take cancer classification accuracy into account without disclosing any biological information of the cancer classification process. Few studies have investigated the biological interpretation of microarray data sets in addition to the model classification accuracy. This research aims to bridge the gap between cancer classification and biological interpretation by improving accuracy performance, and the selected significant genes agree with the findings of relevant biomedical studies. 

This paper proposes a hybrid model based on three different machine learning techniques, including the commonly used ensemble classification methods of gradient boosting [10], an extremely efficient ML algorithm that produces a strong learner in the form of an ensemble of weak learners/models. Furthermore, to optimize the hyperparameters of machine learning algorithms, distributed hyperparameter optimization [11] is one of the most efficient methods (per function evaluation) that is utilized for parameter optimization. Furthermore, minimum redundancy maximum relevance (mrmr) [12] is a particularly fast feature selection method that can be used to find a set of relevant and complementary features. Our model was assessed using three different machine learning classifiers: random forest (RF), naive Bayes (NB), and support vector machines (SVM). 

The experiments show that the proposed model substantially reduces the number of genes required for classification and improves classifier accuracy. Additionally, the proposed hybrid model’s selected genes (features) are biologically interpreted, and the biological interpretation coincides with the findings of relevant biomedical studies. 

The main contributions of this work are:

1. The proposal of a novel ensemble classifier to ensure that the genes selected in our model are biologically interpreted. On top of that, the results are also satisfactory and in line with pertinent biomedical studies.
2. The identification of relevant and non-redundant genes for the biological context by ensemble mRMRs, allowing for enhanced biological interpretations.
3. The analysis of a brain cancer microarray dataset on high-dimensional data using Catboost and XGboost.
4. The optimization of the hyperparameters of the two classifiers using the hyperboot optimizer.
5. The outperformance of Catboost compared to XGboost with regard to the AUC, sensitivity, specificity, and accuracy.

The remainder of the paper is structured as follows: Section 2 briefly reviews related work, while Section 3 reviews some background research that was used in the proposed methodology. Section 4 explains the proposed model, while Section 5 discusses the experimental design, findings, and discussion. Finally, Section 6 concludes the whole paper by summarizing the contributions of the paper.

## 2. Literature Review

In recent years, multiple pieces of research have used a wide range of machine learning methods for the classification, diagnosis, and treatment of cancer disease. BU-Net was developed by Rehman [13] and others to segment and classify brain tumor regions. Their model with a modified encoder–decoder architecture was proposed for segmenting brain tumors. Tests of the proposed BU-Net architecture were conducted on BraTS 2017 and 2018. BU-Net proved to be a significant improvement over the baseline U-Net architecture and other existing segmentation models. Radiology mMRI imaging sub-regions can affect tumor localization, so researchers suggested a deep learning method [14] that considers the uncertainty of the tumor location. Then, they classified the tumor segments into subtypes using a conventional 3D convolutional neural network (CNN). Performance was measured by widely used measures such as the dice score coefficient, the Hausdorff distance at percentile 95 (HD95), classification accuracy, and mean square error. According to the findings, the suggested method can accurately segment tumors and predict survival rates.

Bashir et al. [15] used a fusion of five classifiers: naive Bayes, decision tree using Gini index, decision tree using information gain, support vector machines, and memory-based learner, to diagnose breast cancer. The weighted vote-based ensemble technique was then used to make the final prediction. Several preprocessing and feature selection methods were also used on four breast cancer datasets to improve the prediction accuracy. The proposed ensemble classifiers achieved remarkable results, with average accuracy, precision, and recall of 85.23%, 86.18%, and 76.68%. However, small datasets were used to test the performance of the model. Applying the proposed ensemble on a large dataset with many features may lead to computational instability.

To overcome the dimensionality problem, Kumar et al. [16] introduced the ANOVA, Kruskal–Wallis, and Friedman tests as examples of statistical methods (tests) based on MapReduce to select relevant features. The MapReduce-based proximal support vector machine (mrPSVM) classifier was also applied to classify the microarray data after feature selection. The Hadoop framework was used to implement these algorithms. Using the microarray datasets of different dimensions, a comparative study of these feature selection methodologies was performed. The experimental results showed that an ensemble of the mrPSVM classifier and various feature selection methods produced higher accuracy than other models. Thus, the proposed model successfully handled big data, but it could only interpret biological microarray data. Finally, a two-phase hybrid model for cancer classification was proposed (iBPSO) by Jain et al. [17] that consisted of correlation-based feature selection (CFS) and improved-binary particle swarm optimization. The proposed model uses the naive Bayes classifier to select a low-dimensional collection of prognostic genes to identify biological samples of binary and multi-class cancers. The model was evaluated and tested on eleven benchmark microarray datasets. The findings of the experiments showed that the model outperformed several well-known approaches in terms of classification accuracy and the number of selected genes.

Pradana et al. [18] introduce an approach that used binary particle swarm optimization (BPSO) as a feature selection and C4.5 decision tree as a classifier to investigate cancer diagnosis based on microarray data. The decision tree rule model requires discretization, which is accomplished by the use of K-Means. Applying BPSO and decision tree showed that the model could successfully find the most significant features and increase the accuracy. The model achieved accuracies of 54% and 99%, respectively, for C4.5 and BPSO. Shukla et al. [19] also proposed a new filter-based gene selection approachto identify highly important genes in microarray gene expression datasets. The proposed approach was evaluated using well-known classification techniques such as support vector machine, naive Bayes, *k*-nearest neighbor, and decision tree on the diffuse large B cell lymphoma (DLBCL) dataset. Experiments confirmed that the proposed model could successfully compete, with excellent predictions concerning the accuracy, precision, sensitivity, F-measure, and ROC value.

Sampathkumar et al. [20] developed a novel cuckoo search with a crossover algorithm that could accurately classify a variety of cancer subtypes. The model was tested on benchmark cancer gene expression, and the results show that CSC outperformed CS and other well-known methods. Kilicarslan et al. [21] used the relief algorithm for dimension reduction and feature ranking. The most important features were then used by support vector machines (SVM) and convolutional neural networks (CNN) [22] for prediction. The experimental results show that the proposed approach could improve the accuracy of SVM and CNN classification methods. Finally, Lee et al. [23] suggested a novel multivariate feature ranking approach to increase gene selection efficiency in microarray data. The proposed method created a new feature ranking method by embedding the formal concept of relevance into a Markov blanket (MB). The results show that the model performs well in high-dimensional microarray data classification.

Given all that has been mentioned so far, all of the algorithms listed above address two major issues: brain cancer classifiers and the curse of dimensionality. However, there is no biological interpretation of the microarray data set discussed in the literature. To the best of our knowledge, this paper is the first paper to provide a consolidated biological interpretation of the results of the proposed work. Table 1 summarizes some of the previous research methods for microarray cancer classification to conclude and review related work.

## 3. Materials and Methods

This section discusses ensemble classification and how to set the hyperparameter values and the minimum redundancy maximum relevance (mRMR) feature selection. The three main components of the proposed method are ensemble classification, hyperparameter optimization, and minimum redundancy maximum relevance (mRMR). First, the ensemble methods use multiple learning algorithms to achieve better predictive performance than if they were used by themselves. Secondly, we have hyperparameter optimization, in which the parameters of the classifier are tuned to find the optimal setting. The third one is minimum redundancy maximum relevance (mRMR), which is a filter-type feature selection method that obtains the best feature set by minimizing the similarities between features and classified variables and by maximizing their correlations

### 3.1. Ensemble Classification

**Figure 1:**
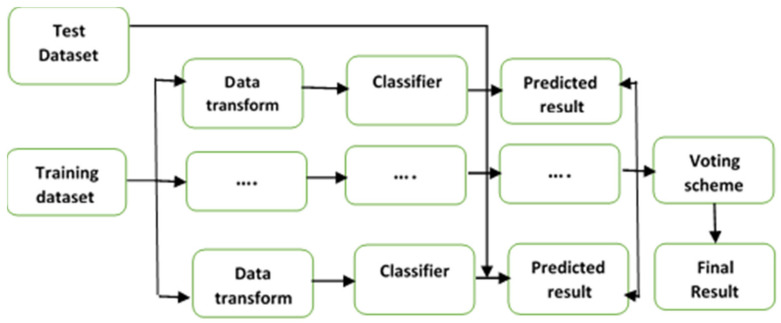
The ensemble classification framework.

Ensemble learning methods use multiple machine learning algorithms to generate weak predictive results based on the features extracted from various data projections. Following this, the results are fused with various voting mechanisms to achieve better results than any constituent algorithm alone [32,33]. Figure 1 depicts the basic concept of a typical ensemble classification model [34], which consists of two steps: (1) generating classification results using multiple weak classifiers and (2) integrating multiple results into a consistency function to obtain the results with voting schemes.

**Figure 2:**
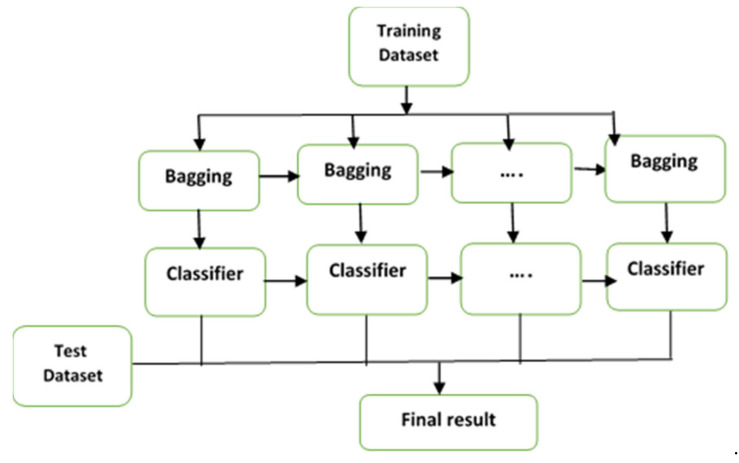
The gradient boosting framework.

The gradient boosting method, which constructs the solution stagewise and solves the overfitting problem by optimizing the loss functions, is one of the most commonly used ensemble classification methods. The main concept of a gradient boosting model is depicted in Figure 2.

XGBoost is an efficient gradient-boosted decision tree algorithm [35]. Gradient boosting is a technique introduced by XGB. The new models are fitted to residuals from previous models, and the combined results are minimized using gradient descent. Yandex released CatBoost [36] in 2017 and demonstrated that it was faster in terms of prediction making, that it was more accurate, and that it was easier to use for categorical data across a series of GBDT tasks based on their benchmark. As a better gradient boosting algorithm, Catboost introduces ordered boosting. Table 2 illustrates the two gradient boosting algorithms.

### 3.2. Hyperparameter Optimization

Hyperopt is a Python library that implements sequential model-based optimization (SMBO) [9]. Hyperopt provides algorithms and software infrastructure to conduct hyperparameter optimization for machine learning algorithms. Hyperopt has an optimization interface that separates a configuration space from an evaluation function that assigns real-valued loss values to points in the configuration space. It works by treating the search of hyperparameters as an optimization task.

Hyperparameter search spaces are typically large multi-dimensional spaces. Hyperopt outperforms grid and random searches, particularly as the search space grows. Within the framework of our proposed model, Hyperopt is used to optimize the settings for the XGBoost and CatBoost hyperparameters. It aims to identify the optimal genes for microarray data analysis and to improve the classification of cancer microarrays.

### 3.3. Minimum Redundancy Maximum Relevance (mRMR) for Feature Selection

Minimum redundancy maximum relevance (mRMR) is a filter-type feature selection method that obtains the best feature set by maximizing the correlation between the features and the classified variables and by minimizing the correlation between features. The classic function enables the collection of appropriate and non-redundant features with ease [37]. In set S, the maximally important and minimally redundant gene i* is given by:

(1) $$i^{*}=argmaX_{i}\in S\;\frac{R_{S}}{Q_{S,i}}$$

Ensemble (mRMR) feature selection implements two ensemble approaches: exhaustive and bootstrap ensemble mRMR. The exhaustive variant of the mRMR heuristic extends it by starting multiple feature selection procedures, with the k>1 being the most relevant feature. Following that, k mRMR solutions are generated in parallel, with the first selected feature being guaranteed to be different. The bootstrap variant resamples the original dataset (with replacement) to generate k bootstraps. Finally, classical mRMR feature selection is performed in parallel for each bootstrapped dataset, resulting in k mRMR solutions.

## 4. The Proposed Hybrid Model

**Figure 3:**
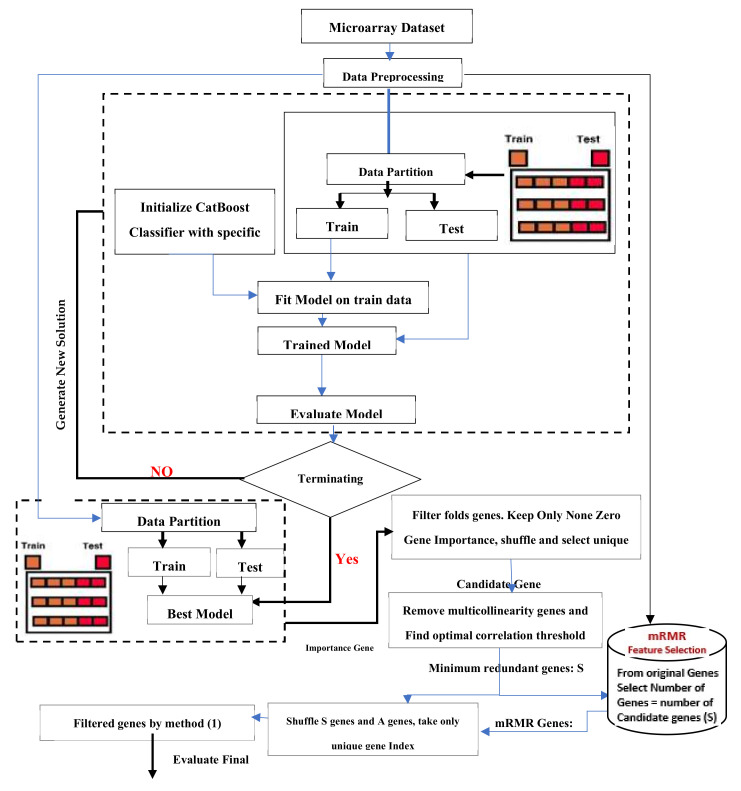
The proposed hybrid model.

This section describes the model (as shown in Figure 3) used for brain cancer classification. The Hyperopt optimizer is used to estimate the optimal values of the CatBoost hyperparameters.

The main process of the model can be defined as:

(i) Preprocessing the dataset (brain cancer microarray). This step is vital to
  - Avoid features in greater numeric ranges dominating those in smaller ranges;
  - Avoid numerical difficulties during calculation;
  - Ensure that each feature is scaled to the range [0, 1].
(ii) The data were partitioned into two sets: The training set is used for the training. The testing set is used to test final model 3, initializing CatBoost with specific solution parameters. Table 3 describes the parameter initialization of the classifiers
(iii) CatBoost is used as a feature selector with 8-fold cross-validations (8 cross-validations of different levels of importance for every gene index). CatBoost calculates the means for each fold.
(iv) By setting a threshold, irrelevant features are then removed. Suppose the score of a gene is above the threshold. In that case, the gene will be selected (as seen in Appendix A, the optimal threshold that offers the maximum accuracy is: 0.84). The genes are shuffled, and unique genes are kept.
(v) The importance value of each gene is registered using a voting process. For example, the gene with index 1 in fold 0 receives an importance value of 1 if the same gene is present in the next fold; then, the gene importance is +1, and so on, for all of the 8-fold cross-validations. After this was applied, voting is conducted 50 genes (six of the filtered genes are genes with an importance >8.

## 5. Results and Discussion

In this section, we present the findings of the various experiments to judge the performance of the proposed hybrid model. A PC with the following features was used to test the proposed hybrid model: Intel(R) Core (TM) i5-7500 CPU with a 32-bit operating system, 4 GB RAM, and the Windows 7 operating system as well as the NumPy, SciPy, Pandas, Keras, and Matplotlib frameworks and Python 2.7 programming language.

### 5.1. Datasets

Molecular profiles from 28 patient samples were analyzed (data set A: medulloblastomas, CNS AT/RTs, renal and extrarenal rhabdoid tumors, supratentorial PNETs, and normal human cerebella). In addition, an analysis of frozen specimen RNA with oligonucleotide microarrays containing probes for 1070 genes was conducted. Gene expression data are available in the Supplementary Information [30].

*k*-fold tests were used in the majority of previous experiments. *k*-fold verification works to find crossed validity by randomly dividing data into k subsets of (approximately) equal size and *k*-times. As a result, it will run several times, with one subset serving as a test group and the other subset, *k*-1, serving as a training group (see Figure 3). The mean of the *k*-fold results can then be averaged to present a single evaluation. In our experiments, we used eight-fold cross-validation to evaluate the outcomes of the proposed hybrid model, and the results are represented as an average standard deviation. Furthermore, the total number of iterations in all of the experiments was 30. In our experiments, we used three evaluation methods [38]: specificity (Spec.), sensitivity (Sen.), and area under the curve (AUC) (AUC).

Sensitivity, as calculated by $S=TP/\left(TP+FN\right),$ is the likelihood that a diseased person is recognized as diseased through the test, where TN is the true negatives number, and TP is the true positives number, and N is the false negative. **Specificity** is the likelihood that a person without the illness is defined by the ((TEST) as non-diseased (or healthy). It is described as $TNR=\left(TN\right)/\;\left(TN+FP\right)$, where FP implies the number of false positives, and TN is the number of true negatives. The **AUC** shows the area under the receiver operating characteristics (ROC) curve, calculated as AUC=(1+TPR−FPR)/2.

### 5.2. Experiment 1: Comparing Performance of Optimized (CatBoost and XGBoost) with the Proposed Hybrid Model

**Figure 4:**
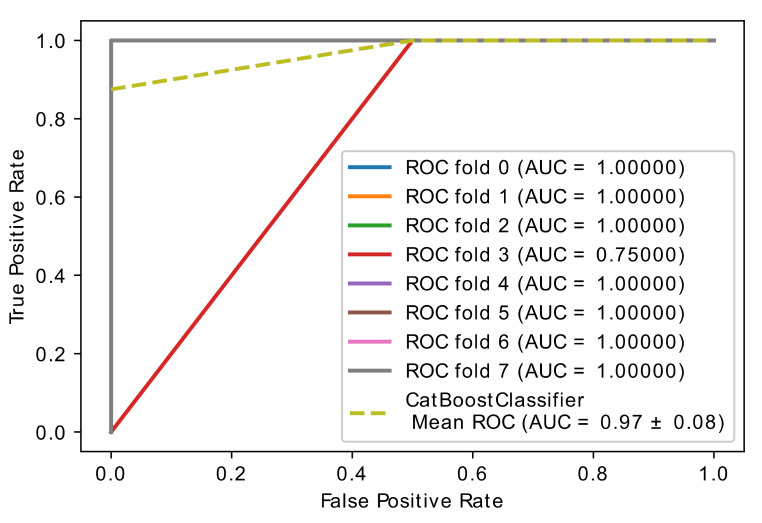
Accuracy curve obtained using the hybrid model with optimized CatBoost classifier.

**Figure 5:**
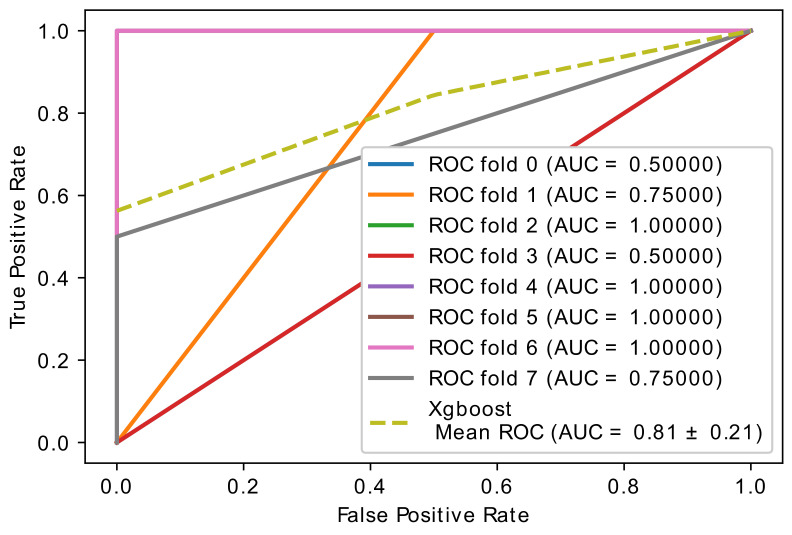
Accuracy curve obtained using the hybrid model with optimized XGBoost classifier.

This experiment compares optimized (CatBoost and XGBoost) classifiers in the proposed hybrid model to achieve the best hyperparameter of the two classifiers. The Hyperopt optimizer is used. Table 3 lists the hyperparameters settings values of XGBoost and CatBoost and the range of each parameter. The experimental results are shown in Table 4 and Figure 4 and Figure 5. The most striking results are better results for the optimized CatBoost classifier than the optimized XGBoost with cross-validation 5, 6, 8, and 10 in the AUC, Sen, and Spec results.

From the data in Figure 4 and Figure 5, it is apparent that CatBoost classifier has the best accuracy, 0.97 ± 0.08, with 8-fold cross-validation and the best AUC=0.97 ± 0.08, Sen=0.94 ±0.17, and Spec=1.00 ± 0.00 compared to the XGBoost classifier, which had an accuracy of 0.80 ± 0.21, and where the AUC=0.80 ± 0.21, Sen=0.80 ± 0.21, and Spec=0.80 ± 0.21. All in all, these results point to CatBoost having a higher performance than XGBoost in the hybrid model we developed. Table 5 shows the training vs. testing performance with 8-fold cross-validation in 28 samples.

### 5.3. Experiment 2: Comparing Performance of CatBoost and Optimized CatBoost Classifier

**Figure 6:**
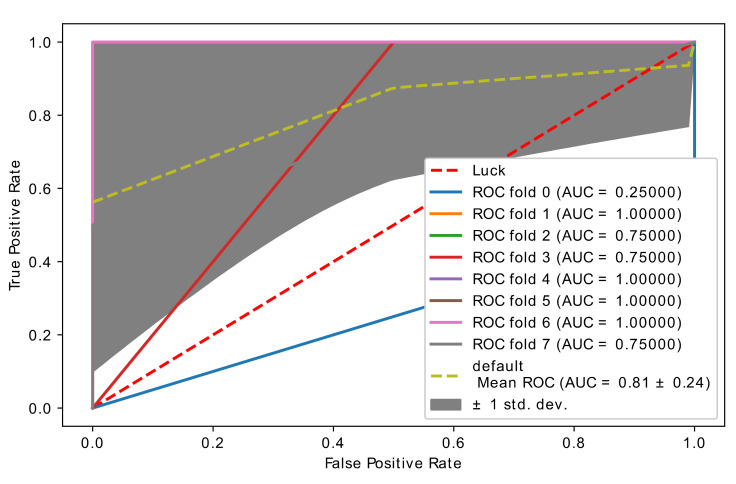
Accuracy curve obtained using the hybrid model with CatBoost classifier.

**Figure 7:**
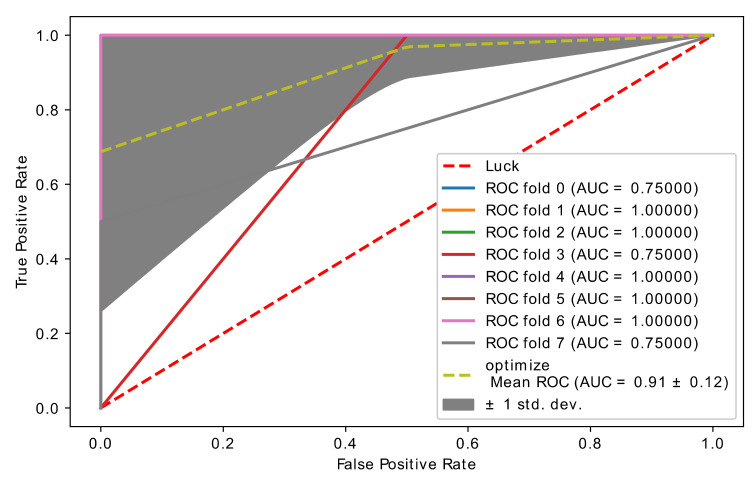
Accuracy curve obtained using the hybrid model with optimized CatBoost classifier.

In this experiment, CatBoost and optimized CatBoost were compared with the original brain cancer microarray data (1070 feature, 28 samples). Table 6 and Figure 6 and Figure 7 show the classification report of the CatBoost and optimized CatBoost classifier with the brain cancer microarray data. The threshold value was 0.84, which is the threshold value with the highest accuracy (see Appendix A) with the final optimal genes (features) selected with our proposed hybrid model. The number of genes selected in each stage of feature selection is as follows:

- Number of non-zero genes importance (every fold).
- (588, 576, 590, 599, 594, 579, 585, 584).
- The number of genes selected by embedded SVM (with Redundant), 980 genes.
- The number of genes selected by embedded SVM (Unique), 671 genes.
- The final number of genes after we applied voting was 50 genes.

Based on the performance metrics in Table 6, optimized CatBoost had higher performance than the CatBoost classifier without optimization. Figure 6 and Figure 7 show the accuracy curve of CatBoost and the optimized CatBoost classifier. The optimized CatBoost classifier had an accuracy of 0.91 ± 0.12, which is higher than that of the CatBoost classifier without optimization, which had an accuracy of 0.81 ± 0.24.

### 5.4. Experiment 3: Comparison of Hybrid Proposed Model Performance by Different Classification

**Figure 8:**
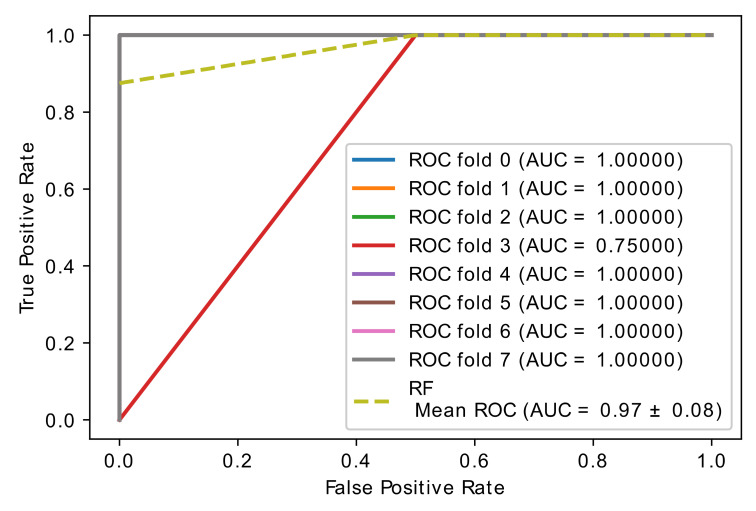
Accuracy curve of random forest classifier.

**Figure 9:**
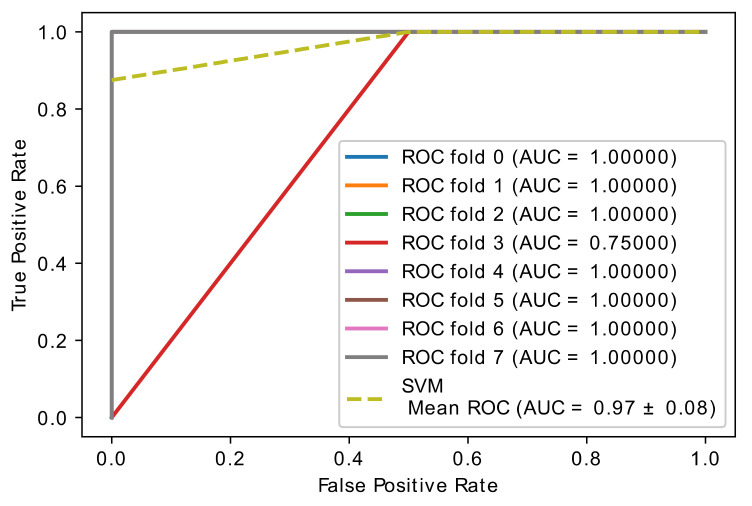
Accuracy curve of SVM classifier.

**Figure 10:**
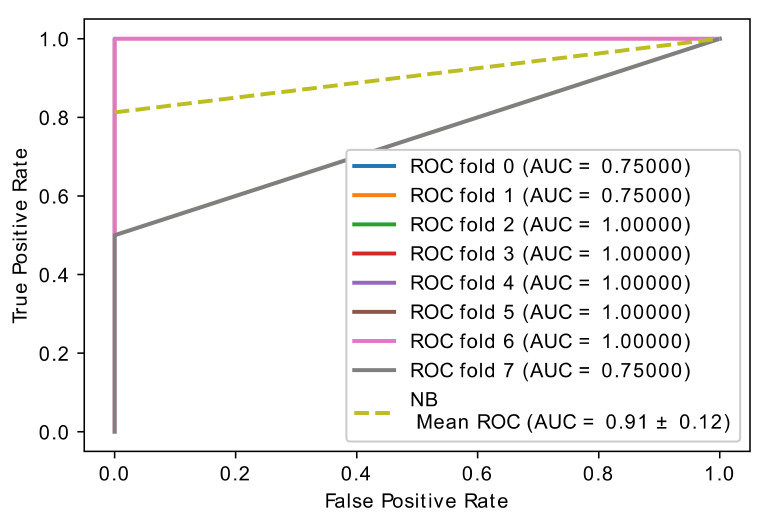
Accuracy curve of NB classifier.

The classic learning algorithms random forest (RF), naive Bayes (NB), and support vector machines (SVM) were used to assess the gene classification accuracy of selected optimal genes by the proposed hybrid model. These learning algorithms were applied to the newly collected dataset, which only included the best genes, and the overall accuracy was calculated. In Figure 8, Figure 9 and Figure 10, the learning accuracy of three classifiers is illustrated using the newly generated gene (feature) set. The proposed hybrid model increased the accuracy of the SVM, RF, and NB classifiers while the accuracy is weighted on brain data set; on the other hand, SVM and RF, with accuracies of (0.97 ± 0.08), achieve equal and higher classification accuracy than the NB (0.91 ± 0.12) classifier.

### 5.5. Biological Interpretation

A subset of genes (features) from the brain cancer data set was biologically interpreted to demonstrate the proposed model’s efficacy in improving critical items such as classification accuracy and for selecting genes with important biological backgrounds. A few classes of important genes derived from microarray technologies were used to diagnose and to provide the prognostic purposes of brain cancer after using the proposed hybrid model’s biological portrait.

The proposed hybrid model aims to determine crucial gene subsets with the maximum amount of accuracy needed to treat a brain cancer patient. In this segment, the selected group of probe sets could be studied by using the web tool DAVID (Database for Annotation, Integrated Discovery, and Visualization) https://david.ncifcrf.gov/list.jsp (accessed on 18 June 2020) [31,39]. Table 7 shows the gene name and gene ID from the Entrez probe set. GO Research Tools: http://www.geneontology.org/GO.tools.microarray, (accessed on 18 June 2020) are generally considered to be the most inclusive and fastest-growing public repository for grouping functionally related genes. Following that, it can be shown that the proposed approach is the most effective way to pick a large group of genes for brain cancer pathway detection and prognosis.

**Figure 11:**
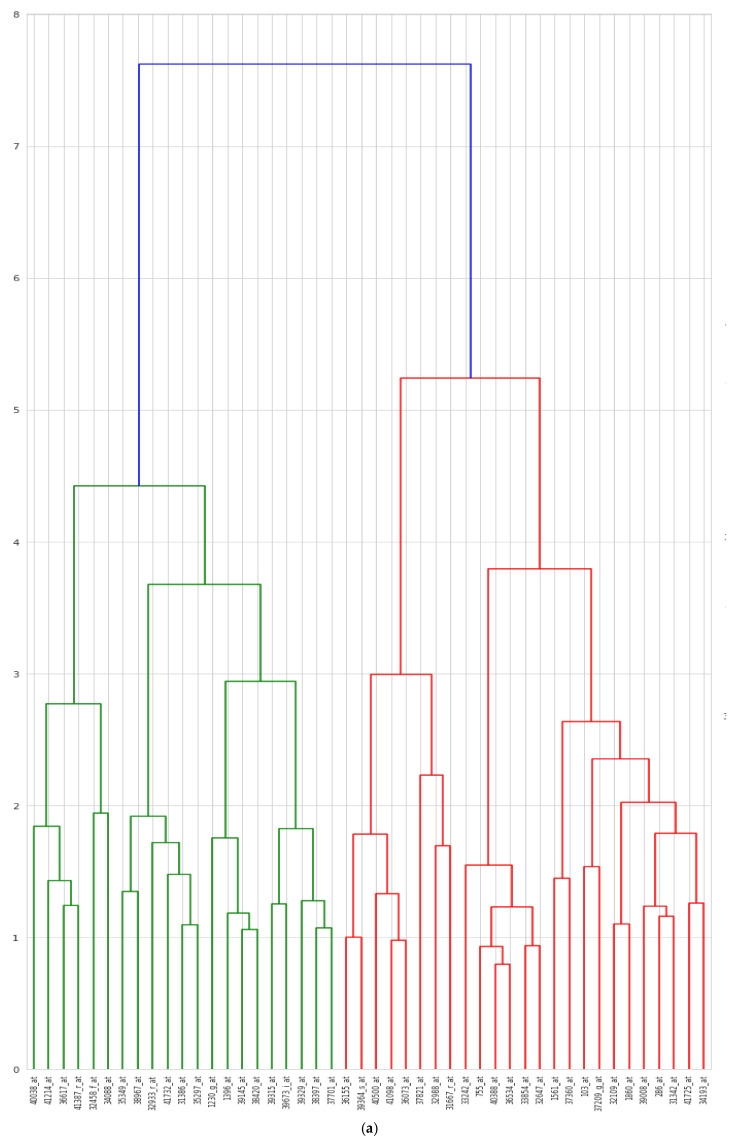

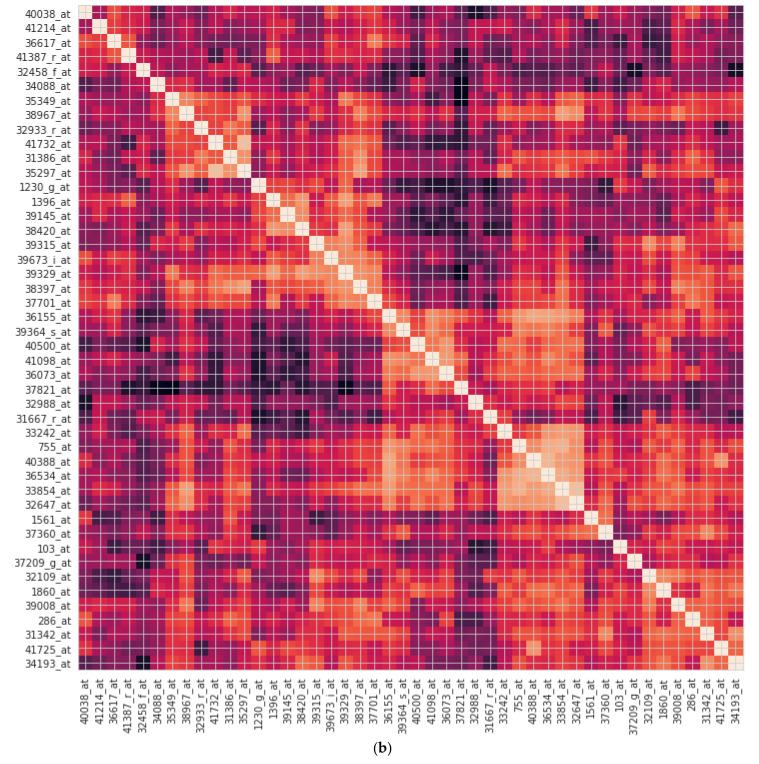
(**a**) Hierarchical clustering dendrogram maps of the genes selected in the proposed hybrid model. (**b**) Heat maps of the genes selected in the proposed hybrid model.

In Figure 11, heat maps representing the frequency of selected features over the cross-validation analysis are used to evaluate the consistency of the selected features over time and to identify genes that are differentially expressed between the two disease classes (cancer class and healthy class). 

**Figure 12:**
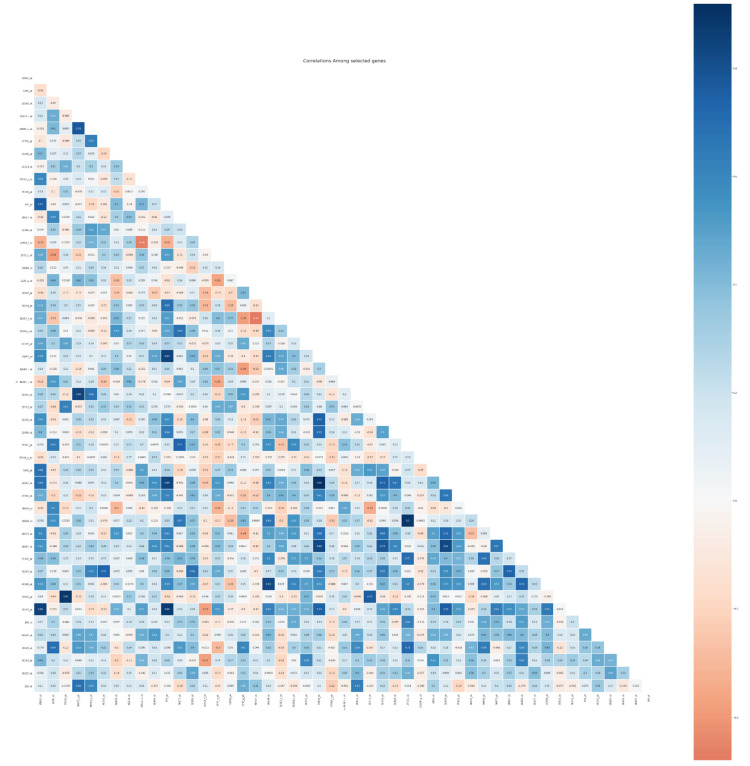
The correlation among the selected genes with the proposed hybrid mode.

Figure 12 shows the correlation among the selected feature using our proposed model with the Catboost classifier. The correlation coefficient has values between −1 to 1. A value closer to 0 implies a weaker correlation (exact 0 implying no correlation). A value closer to 1 implies a stronger positive correlation, and a value closer to −1 implies a stronger negative correlation. We compared the correlation between features and removed one of two features that correlate to >= 0.5, that correlate to =0.5 in the first threshold, and to 0.5 + 0.01 in the second fold of the threshold.

## 6. Conclusions

Brain disorders are becoming a major issue, particularly malignant brain tumors, which significantly impact people’s lives. The brain cancer microarray data have proven to be a complicated classification task due to the small number of samples that have a large number of gene expression levels as features. As part of brain cancer microarray data analysis, the present study proposed an effective and powerful technique for the selection of significant and relevant genes with biomedical relevance. Three distinct techniques were used for classification and prediction (feature selection, optimization, and classification). We used the same dataset and three different algorithms to evaluate the performance of the proposed model (NB, RF, and SVM). The experimental results demonstrate that the proposed hybrid model significantly improves critical items such as classification accuracy and that the selected genes have an important biological background.

Furthermore, selecting optimal genes (features) with biological significance can assist biological researchers in brain cancer treatment. The major contributions of this paper are: (a) The application of Catboost and XGboost on high-dimensional microarray data to create a cancer microarray dataset; (b) the use of the hyperboot optimizer to optimize the hyperparameters of the two classifiers, and the outperformance of the Catboost on XGboost in terms of AUC, Sen, Spec, and accuracy; (c) the selection of genes that are non-redundant and relevant to the biological context using ensemble mRMR, which leads to more detailed biological interpretations. Later, the output of the gene subset was combined with the Catboost-selected features. Then, a voting process was applied to obtain unique, informative genes (features) with high relevance and minimum redundancy; (d) the selected genes in our proposed model were biologically interpreted, and the results agree with the findings of relevant biomedical studies. Developing robustness should be a priority for future work.

## Figures and Tables

**Table 1:** Review of previous studies on the cancer microarray data classification.

Author	Method	Remark	Limitations	Dataset
Bashir, S., Qamar, U., and Khan, F. H. (2015) [15].	(Naïve Bayes, DT-Gini, DT-IG, MBL and SVM)	- A weighted vote-based ensemble fusion of heterogeneous classifiers was introduced for dynamic breast cancer diagnosis.	- Small datasets were used to test the performance of the model. - A small set of features are used to test the proposed model.	- UCI [24] - Wisconsin Clinical [25]
Kumar, M., and Rath, S. K. (2015) [16].	(MrPSVM)	- Data on microarrays are classified using proximal support vector machines (mrPSVMs) based on MapReduce. - Large-scale results were managed using Hadoop.	- No biological interpretation of the microarray data set was discussed.	- Kent Ridge Bio-medical Data Set Repository [26] - National center of Biotechnology Information (NCBI GEO) [27]
Jain, I., Jain, V. K., and Jain, R. (2018) [17]	(CFS) and (iBPSO)	- Hybrid feature selection is proposed for gene selection and cancer classification that combines correlation-based and binary particle swarm optimization. - Tested on eleven benchmark microarray datasets	Biological information on the cancer classification process is not discussed.	- Kent Ridge Bio-medical Data Set Repository [28]
Pradana, A. C., and Aditsania, A. (2019, March) [18]	(BPSO) Decision Tree C4.5)	- Introduced binary particle swarm optimization and C4. 5 decision tree for cancer detection based on microarray data classification. - Used Random Forest Ranking (RFR) as filtering methods to order genes	By using filtering methods, some important features may not be included. There is no interpretation of the results.	- Kent Ridge Bio-medical Data Set Repository [29]
Shukla, A. K., and Tripathi, D. (2020) [19]	Spearman’s Correlation (SC) and distributed FS	- Introduced a new filter-based method for gene selection that can select the highly relevant genes for distinguishing tissues from the gene expression dataset	- Biological information is not addressed.	- DLBCL [30,31]

**Table 2:** XGBoost and CatBoost classifiers.

	Base Classifiers	Ways to Prevent Overfitting	The Loss Function for Binary Classification
XGBoost	Regression trees	▪ Row Subsampling ▪ Shrinkage parameter ▪ Column subsampling ▪ Regularization term in the objective function	$L=y\;log\;\left(p\left(x\right)\right)+\left(1-y\right)\;log\;\left(1-p\left(x\right)\right),$ $p\left(x\right)=\frac{1}{1+\exp\;\left(-F\left(x\right)\right)}$
CatBoost	Classification trees	▪ Row Subsampling ▪ Shrinkage parameter ▪ Column subsampling	$L_{i}=-y_{i}\log p_{i}^{\wedge }\left(1-y_{i}\right)log\left(1-p_{i}^{\wedge }\right)$

**Table 3:** Parameter initialization.

XGBoost Classifier		CatBoost Classifier	
Hyperparameters	Range	Hyperparameters	Range
iterations	[1, 500]	n_estimators	[50, 900]
depth	[1, 16]	max_depth	[1, 12]
subsample	[0.5, 1]	m_child_weight	[1, 6]
rsm	[0.75, 1.0]	gamma	[0.5, 1]
learning_rate	[−3.0, −0.7]	subsample	[0.5, 1]
l2_leaf_reg	[1, 10]	learning_rate	[log(0.001), log(0.3)]
random_strength	[1 × 10^−9^, 10]	colsample_bytree	[0.5, 1]
bagging_temperature	[0.0, 1.0]		
scale_pos_weight	[0.01, 1.0]		

**Table 4:** Comparing performance of optimized (XGboost and Catboost) in terms of AUC, Sen., and Spec.

Cross Validation (CV)	XGBoost Classifier			CatBoost Classifier		
	AUC	Sen	Spec	AUC	Sen	Spec
Cv = 5	0.80 ± 0.16	0.80 ± 0.16	0.80 ± 0.16	0.87 ± 0.07	0.80 ± 0.16	0.93 ± 0.13
Cv = 6	0.83 ± 0.13	0.75 ± 0.26	0.93 ± 0.19	0.89 ± 0.11	0.86 ± 0.20	0.92 ± 0.19
Cv = 8	0.81 ± 0.16	0.81 ± 0.24	0.81 ± 0.24	0.91 ± 0.12	0.88± 0.21	0.93 ± 0.16
Cv = 10	0.75 ± 0.29	0.75 ± 0.33	0.75 ± 0.40	0.88 ± 0.17	0.85 ± 0.23	0.90 ± 0.03

**Table 5:** Training vs. testing performance of optimized Catboost with 8-fold cross-validation.

Fold Number	Optimized CatBoost Classifier	
	Train Accuracy	Test Accuracy
1	1.00	0.750
2	1.00	1.00
3	1.00	0.750
4	1.00	1.00
5	1.00	1.00
6	1.00	1.00
7	1.00	1.00
8	1.00	1.00

**Table 6:** Classification report for CatBoost and optimized CatBoost.

Brain Cancer Dataset	CatBoost				Optimized CatBoost			
	Precision	Recall	f1-Score	Support	Precision	Recall	f1-Score	Support
0.0	0.85	0.79	0.81	14	0.92	0.79	0.85	14
1.0	0.80	0.86	0.83	14	0.81	0.93	0.87	14
accuracy			0.82	14			0.82	14
Macro avg	0.82	0.82	0.82	28	0.82	0.82	0.82	28
Weighted avg	0.82	0.82	0.82	28	0.82	0.82	0.82	28

**Table 7:** Gene accession number and gene description of the selected genes of brain cancer by the proposed hybrid model.

Prob set	Gene ID	Gene Name	Diagnostic Marker	Prognostic Marker	Overexpression	Down Expression
1860_at	1860	Tumor protein p53 binding protein 2(TP53BP2)		Unfavorable	+	
286_at	286	Histone cluster 2 H2A family member a4(HIST2H2AA4)	Yes		+	
31667_r_at	31667_r	Nuclear receptor subfamily 2 group E member 3(NR2E3)	Yes		+	
33242_at	33242	TSR2, ribosome maturation factor(TSR2)	Yes		+	
34088_at	34088	Neurexophilin 4(NXPH4)		Unfavorable	+	
37055_at	37055	ETS variant 1(ETV1)		Unfavorable	+	
37701_at	37701	Regulator of G-protein signaling 2(RGS2)		Unfavorable	+	
40388_at	40388	DLG associated protein 1(DLGAP1)		Unfavorable		
41098_at	41098	Dishevelled associated activator of morphogenesis 2(DAAM2)		+		
1972_s_at	1972_s	Microtubule associated protein 2(MAP2)		Unfavorable	+	
32647_at	32647	Vesicle transport through interaction with t-SNAREs 1B(VTI1B)	Yes			+
36073_at	36073	Necdin, MAGE family member(NDN)		Unfavorable	+	
37360_at	37360	Lymphocyte antigen 6 complex, locus E(LY6E)	Yes		+	
38420_at	38420	Collagen type V alpha 2 chain(COL5A2)		Unfavorable	+	
39673_i_at	39673_i	Extracellular matrix protein 2(ECM2)		Unfavorable		+
41387_r_at	41387_r	Lysine demethylase 6B(KDM6B)		Unfavorable	+	
41407_at	41407	MicroRNA 1236(MIR1236)		Unfavorable	+	
41725_at	41725	Casein kinase 1 gamma 2(CSNK1G2)		Unfavorable	+	
41732_at	41732	BolA family member 2(BOLA2)		Favorable	+	
103_at	103	Thrombospondin 4(THBS4)		Unfavorable		+
1230_g_at	1230_g	Myotubularin related protein 11(MTMR11)	Yes			+
1396_at	1396	Insulin like growth factor binding protein 5(IGFBP5)		Unfavorable	+	
32988_at	32988	Chloride voltage-gated channel Ka(CLCNKA)		Unfavorable	+	
33854_at	33854	ATPase H+ transporting V1 subunit D(ATP6V1D)		Unfavorable	+	
37209_g_at	37209_g	Phosphoserine phosphatase(PSPH)		Unfavorable	+	
35297_at	35297	NADH:ubiquinone oxidoreductase subunit AB1(NDUFAB1)		Unfavorable	+	
36155_at	36155	SPARC/osteonectin, cwcv and kazal-like domains proteoglycan 2(SPOCK2)		Favorable	+	
36534_at	36534	DIX domain containing 1(DIXDC1)		Unfavorable	+	
36617_at	36617	Inhibitor of DNA binding 1, HLH protein(ID1)		Unfavorable		+
38440_s_at	38440_s	Armadillo repeat containing, X-linked 6(ARMCX6)		Unfavorable	+	
39315_at	39315	Angiopoietin 1(ANGPT1)		Unfavorable	+	
39364_s_at	39364_s	Protein phosphatase 1 regulatory subunit 3C(PPP1R3C)		Unfavorable		
39512_s_at	39512_s	Inositol polyphosphate-4-phosphatase type I A(INPP4A)		+		
39850_at	39850	Ankyrin 2(ANK2)		Unfavorable	+	
755_at	755	Inositol 1,4,5-trisphosphate receptor type 1(ITPR1)				
31386_at	31386	Immunoglobulin kappa variable 1/OR2-118 (IGKV1OR2-118) (pseudogene)		Unfavorable	+	
33580_r_at	33580_r	Galanin receptor 3(GALR3)			+	
34193_at	34193	Cell adhesion molecule L1 like(CHL1)		Unfavorable	+	
35349_at	35349	COP9 signalosome subunit 3(COPS3)		Unfavorable		+
35719_at	35719	PH domain and leucine rich repeat protein phosphatase 1(PHLPP1)		Unfavorable	+	
38967_at	38967	Chromosome 14 open reading frame 2(C14orf2)		Unfavorable	+	
39329_at	39329	Actinin alpha 1(ACTN1)	yes	Unfavorable	+	
41530_at	41530	Acetyl-CoA acyltransferase 2(ACAA2)		Favorable	+	
38397_at	38397	DNA polymerase delta 4, accessory subunit(POLD4)		Unfavorable		
39008_at	39008	Ceruloplasmin(CP)				
40767_at	40767	Tissue factor pathway inhibitor(TFPI)		Unfavorable	+	
41214_at	41214	Ribosomal protein S4, Y-linked 1(RPS4Y1)		Unfavorable	+	
31342_at	31342	Polypeptide N-acetylgalactosaminyltransferase 2(GALNT2)		Unfavorable	+	
32109_at	32109	FXYD domain-containing ion transport regulator 1(FXYD1)	yes	Unfavorable	+	
32458_f_at	32458_f	Proline rich protein BstNI subfamily 4(PRB4)		Unfavorable	+	

## Data Availability

Data available upon request.

## Appendix A

**Table:**

Threshold (28: 1068)	SVM				Random Forest				Naive Bayes				CatBoost			
	Accuracy	Spec	SEN	AUC	Accuracy	Spec	SEN	AUC	Accuracy	Spec	SEN	AUC	Accuracy	Spec	SEN	AUC
0.5	0.91 ± 0.17	1.00 ± 0.00	0.81 ± 0.35	0.91 ± 0.17	0.97 ± 0.08	1.00 ± 0.00	0.94 ± 0.17	0.97 ± 0.08	1.00 ± 0.00	1.00 ± 0.00	1.00 ± 0.00	1.00 ± 0.00	0.91 ± 0.12	0.94 ± 0.17	0.88 ± 0.22	0.91 ± 0.12
0.51	0.91 ± 0.17	1.00 ± 0.00	0.81 ± 0.35	0.91 ± 0.17	0.97 ± 0.08	0.94 ± 0.17	1.00 ± 0.00	0.97 ± 0.08	1.00 ± 0.00	1.00 ± 0.00	1.00 ± 0.00	1.00 ± 0.00	0.91 ± 0.12	0.94 ± 0.17	0.88 ± 0.22	0.91 ± 0.12
0.52	0.91 ± 0.17	1.00 ± 0.00	0.81 ± 0.35	0.91 ± 0.17	0.91 ± 0.17	0.88 ± 0.33	0.94 ± 0.17	0.91 ± 0.17	1.00 ± 0.00	1.00 ± 0.00	1.00 ± 0.00	1.00 ± 0.00	0.91 ± 0.12	0.94 ± 0.17	0.88 ± 0.22	0.91 ± 0.12
0.53	0.91 ± 0.17	1.00 ± 0.00	0.81 ± 0.35	0.91 ± 0.17	0.94 ± 0.11	1.00 ± 0.00	0.88 ± 0.22	0.94 ± 0.11	1.00 ± 0.00	1.00 ± 0.00	1.00 ± 0.00	1.00 ± 0.00	0.91 ± 0.12	0.94 ± 0.17	0.88 ± 0.22	0.91 ± 0.12
0.54	0.91 ± 0.17	1.00 ± 0.00	0.81 ± 0.35	0.91 ± 0.17	0.97 ± 0.08	1.00 ± 0.00	0.94 ± 0.17	0.97 ± 0.08	1.00 ± 0.00	1.00 ± 0.00	1.00 ± 0.00	1.00 ± 0.00	0.91 ± 0.12	0.94 ± 0.17	0.88 ± 0.22	0.91 ± 0.12
0.55	0.91 ± 0.17	1.00 ± 0.00	0.81 ± 0.35	0.91 ± 0.17	0.93 ± 0.13	1.00 ± 0.00	0.88 ± 0.22	0.94 ± 0.11	1.00 ± 0.00	1.00 ± 0.00	1.00 ± 0.00	1.00 ± 0.00	0.91 ± 0.12	0.94 ± 0.17	0.88 ± 0.22	0.91 ± 0.12
0.56	0.91 ± 0.17	1.00 ± 0.00	0.81 ± 0.35	0.91 ± 0.17	0.97 ± 0.08	1.00 ± 0.00	0.94 ± 0.17	0.97 ± 0.08	1.00 ± 0.00	1.00 ± 0.00	1.00 ± 0.00	1.00 ± 0.00	0.91 ± 0.12	0.94 ± 0.17	0.88 ± 0.22	0.91 ± 0.12
0.57	0.91 ± 0.17	1.00 ± 0.00	0.81 ± 0.35	0.91 ± 0.17	0.94 ± 0.11	0.94 ± 0.17	0.94 ± 0.17	0.94 ± 0.11	1.00 ± 0.00	1.00 ± 0.00	1.00 ± 0.00	1.00 ± 0.00	0.91 ± 0.12	0.94 ± 0.17	0.88 ± 0.22	0.91 ± 0.12
0.58	0.93 ± 0.13	1.00 ± 0.00	0.88 ± 0.22	0.94 ± 0.11	0.88 ± 0.12	0.94 ± 0.17	0.81 ± 0.24	0.88 ± 0.12	0.97 ± 0.08	0.94 ± 0.17	1.00 ± 0.00	0.97 ± 0.08	0.91 ± 0.12	0.94 ± 0.17	0.88 ± 0.22	0.91 ± 0.12
0.59	0.97 ± 0.08	1.00 ± 0.00	0.94 ± 0.17	0.97 ± 0.08	0.91 ± 0.12	0.94 ± 0.17	0.88 ± 0.22	0.91 ± 0.12	1.00 ± 0.00	1.00 ± 0.00	1.00 ± 0.00	1.00 ± 0.00	0.91 ± 0.12	0.94 ± 0.17	0.88 ± 0.22	0.91 ± 0.12
0.6	0.97 ± 0.08	1.00 ± 0.00	0.94 ± 0.17	0.97 ± 0.08	0.91 ± 0.12	0.94 ± 0.17	0.88 ± 0.22	0.91 ± 0.12	1.00 ± 0.00	1.00 ± 0.00	1.00 ± 0.00	1.00 ± 0.00	0.91 ± 0.12	0.94 ± 0.17	0.88 ± 0.22	0.91 ± 0.12
0.61	0.97 ± 0.08	1.00 ± 0.00	0.94 ± 0.17	0.97 ± 0.08	0.86 ± 0.19	0.88 ± 0.22	0.88 ± 0.22	0.88 ± 0.18	0.97 ± 0.08	0.94 ± 0.17	1.00 ± 0.00	0.97 ± 0.08	0.90 ± 0.14	1.00 ± 0.00	0.81 ± 0.24	0.91 ± 0.12
0.62	0.97 ± 0.08	1.00 ± 0.00	0.94 ± 0.17	0.97 ± 0.08	0.90 ± 0.14	0.94 ± 0.17	0.88 ± 0.22	0.91 ± 0.12	0.97 ± 0.08	0.94 ± 0.17	1.00 ± 0.00	0.97 ± 0.08	0.90 ± 0.14	1.00 ± 0.00	0.81 ± 0.24	0.91 ± 0.12
0.63	0.97 ± 0.08	1.00 ± 0.00	0.94 ± 0.17	0.97 ± 0.08	0.83 ± 0.18	0.88 ± 0.22	0.81 ± 0.24	0.84 ± 0.17	0.97 ± 0.08	0.94 ± 0.17	1.00 ± 0.00	0.97 ± 0.08	0.94 ± 0.11	1.00 ± 0.00	0.88 ± 0.22	0.94 ± 0.11
0.64	0.97 ± 0.08	1.00 ± 0.00	0.94 ± 0.17	0.97 ± 0.08	0.97 ± 0.08	1.00 ± 0.00	0.94 ± 0.17	0.97 ± 0.08	0.97 ± 0.08	1.00 ± 0.00	0.94 ± 0.17	0.97 ± 0.08	0.94 ± 0.11	1.00 ± 0.00	0.88 ± 0.22	0.94 ± 0.11
0.65	0.94± 0.11	1.00 ± 0.00	0.88 ± 0.22	0.94 ± 0.11	0.97 ± 0.08	1.00 ± 0.00	0.94 ± 0.17	0.97 ± 0.08	1.00 ± 0.00	1.00 ± 0.00	1.00 ± 0.00	1.00 ± 0.00	0.91 ± 0.12	0.94 ± 0.17	0.88 ± 0.22	0.91 ± 0.12
0.66	0.97 ± 0.08	1.00 ± 0.00	0.94 ± 0.17	0.97 ± 0.08	0.97 ± 0.08	1.00 ± 0.00	0.94 ± 0.17	0.97 ± 0.08	0.97 ± 0.08	0.94 ± 0.17	1.00 ± 0.00	0.97 ± 0.08	0.94 ± 0.11	1.00 ± 0.00	0.88 ± 0.22	0.94 ± 0.11
0.67	0.97 ± 0.08	1.00 ± 0.00	0.94 ± 0.17	0.97 ± 0.08	0.94 ± 0.17	0.94 ± 0.17	0.94 ± 0.17	0.94 ± 0.17	0.97 ± 0.08	0.94 ± 0.17	1.00 ± 0.00	0.97 ± 0.08	0.94 ± 0.17	1.00 ± 0.00	0.97 ± 0.08	0.94 ± 0.11
0.68	0.97 ± 0.08	1.00 ± 0.00	0.94 ± 0.17	0.97 ± 0.08	0.94 ± 0.17	0.94 ± 0.17	0.94 ± 0.17	0.94 ± 0.17	0.97 ± 0.08	0.94 ± 0.17	1.00 ± 0.00	0.97 ± 0.08	0.94 ± 0.11	1.00 ± 0.00	0.88 ± 0.22	0.94 ± 0.11
0.69	0.97 ± 0.08	1.00 ± 0.00	0.94 ± 0.17	0.97 ± 0.08	0.91 ± 0.17	0.94 ± 0.17	0.88 ± 0.22	0.91 ± 0.17	0.97 ± 0.08	0.94 ± 0.17	1.00 ± 0.00	0.97 ± 0.08	0.94 ± 0.11	1.00 ± 0.00	0.88 ± 0.22	0.94 ± 0.11
0.7	0.94 ± 0.11	1.00 ± 0.00	0.88 ± 0.22	0.94 ± 0.11	0.91 ± 0.17	0.94 ± 0.17	0.88 ± 0.22	0.91 ± 0.17	0.91 ± 0.12	0.81 ± 0.24	1.00 ± 0.00	0.91 ± 0.12	0.94 ± 0.11	1.00 ± 0.00	0.88 ± 0.22	0.94 ± 0.11
0.71	0.94 ± 0.11	1.00 ± 0.00	0.88 ± 0.22	0.94 ± 0.11	0.91 ± 0.17	0.94 ± 0.17	0.88 ± 0.22	0.91 ± 0.17	0.93 ± 0.13	0.88 ± 0.22	1.00 ± 0.00	0.94 ± 0.11	0.91 ± 0.12	0.94 ± 0.17	0.81 ± 0.24	0.91 ± 0.12
0.72	0.94 ± 0.11	1.00 ± 0.00	0.88 ± 0.22	0.94 ± 0.11	0.97 ± 0.08	1.00 ± 0.00	0.94 ± 0.17	0.97 ± 0.08	0.90 ± 0.14	0.88 ± 0.22	0.94 ± 0.17	0.91 ± 0.12	0.91 ± 0.12	1.00 ± 0.00	0.81 ± 0.24	0.91 ± 0.12
0.73	0.94 ± 0.11	1.00 ± 0.00	0.88 ± 0.22	0.94 ± 0.11	0.94 ± 0.17	0.94 ± 0.17	0.94 ± 0.17	0.94 ± 0.17	0.90 ± 0.14	0.88 ± 0.22	0.94 ± 0.17	0.91 ± 0.12	0.91 ± 0.12	1.00 ± 0.00	0.81 ± 0.24	0.91 ± 0.12
0.74	0.97 ± 0.08	1.00 ± 0.00	0.94 ± 0.17	0.97 ± 0.08	0.91 ± 0.17	0.94 ± 0.17	0.88 ± 0.22	0.91 ± 0.17	0.93 ± 0.13	0.88 ± 0.22	1.00 ± 0.00	0.94 ± 0.11	0.94 ± 0.11	1.00 ± 0.00	0.88 ± 0.22	0.94 ± 0.11
0.75	0.97 ± 0.08	1.00 ± 0.00	0.94 ± 0.17	0.97 ± 0.08	0.97 ± 0.08	1.00 ± 0.00	0.94 ± 0.17	0.97 ± 0.08	0.90 ± 0.14	0.88 ± 0.22	0.94 ± 0.17	0.91 ± 0.12	0.94 ± 0.11	1.00 ± 0.00	0.88 ± 0.22	0.94 ± 0.11
0.76	0.97 ± 0.08	1.00 ± 0.00	0.94 ± 0.17	0.97 ± 0.08	0.94 ± 0.17	0.94 ± 0.17	0.94 ± 0.17	0.94 ± 0.17	0.80 ± 0.21	0.75 ± 0.35	0.88 ± 0.22	0.81 ± 0.21	0.94 ± 0.11	1.00 ± 0.00	0.88 ± 0.22	0.94 ± 0.11
0.77	0.97 ± 0.08	1.00 ± 0.00	0.94 ± 0.17	0.97 ± 0.08	0.93 ± 0.13	0.94 ± 0.17	0.94 ± 0.17	0.94 ± 0.11	0.80 ± 0.21	0.75 ± 0.35	0.88 ± 0.22	0.81 ± 0.21	0.94 ± 0.11	1.00 ± 0.00	0.88 ± 0.22	0.94 ± 0.11
0.78	0.97 ± 0.08	1.00 ± 0.00	0.94 ± 0.17	0.97 ± 0.08	0.86 ± 0.19	0.88 ± 0.22	0.88 ± 0.22	0.88 ± 0.18	0.83 ± 0.18	0.75 ± 0.35	0.94 ± 0.17	0.84 ± 0.17	0.94 ± 0.11	1.00 ± 0.00	0.88 ± 0.22	0.94 ± 0.11
0.79	1.00 ± 0.00	1.00 ± 0.00	1.00 ± 0.00	1.00 ± 0.00	0.90 ± 0.19	0.88 ± 0.22	0.94 ± 0.17	0.91 ± 0.17	0.83 ± 0.18	0.75 ± 0.35	0.94 ± 0.17	0.84 ± 0.17	0.94 ± 0.11	1.00 ± 0.00	0.88 ± 0.22	0.94 ± 0.11
0.8	1.00 ± 0.00	1.00 ± 0.00	1.00 ± 0.00	1.00 ± 0.00	0.94 ± 0.11	1.00 ± 0.00	0.88 ± 0.22	0.94 ± 0.11	0.86 ± 0.14	0.75 ± 0.35	0.94 ± 0.17	0.84 ± 0.17	0.94 ± 0.11	1.00 ± 0.00	0.88 ± 0.22	0.94 ± 0.11
0.81	1.00 ± 0.00	1.00 ± 0.00	1.00 ± 0.00	1.00 ± 0.00	0.97 ± 0.08	1.00 ± 0.00	0.94 ± 0.17	0.97 ± 0.08	0.83 ± 0.19	0.81 ± 0.24	0.94 ± 0.17	0.88 ± 0.18	0.90 ± 0.14	0.94 ± 0.17	0.88 ± 0.22	0.91 ± 0.12
0.82	1.00 ± 0.00	1.00 ± 0.00	1.00 ± 0.00	1.00 ± 0.00	0.93 ± 0.13	0.94 ± 0.17	0.94 ± 0.17	0.94 ± 0.11	0.86 ± 0.19	0.81 ± 0.24	0.94 ± 0.17	0.88 ± 0.18	0.90 ± 0.14	0.94 ± 0.17	0.88 ± 0.22	0.91 ± 0.12
0.83	1.00 ± 0.00	1.00 ± 0.00	1.00 ± 0.00	1.00 ± 0.00	0.97 ± 0.08	1.00 ± 0.00	0.94 ± 0.17	0.97 ± 0.08	0.90 ± 0.14	0.81 ± 0.24	1.00 ± 0.00	0.91 ± 0.12	0.93 ± 0.13	0.94 ± 0.17	0.94 ± 0.17	0.94 ± 0.11
0.84	1.00 ± 0.00	1.00 ± 0.00	1.00 ± 0.00	1.00 ± 0.00	0.97 ± 0.08	1.00 ± 0.00	0.94 ± 0.17	0.97 ± 0.08	0.90 ± 0.14	0.81 ± 0.24	1.00 ± 0.00	0.91 ± 0.12	0.97 ± 0.08	1.00 ± 0.00	0.94 ± 0.17	0.97 ± 0.08
0.85	0.97 ± 0.08	1.00 ± 0.00	0.94 ± 0.17	0.97 ± 0.08	0.93 ± 0.13	0.94 ± 0.17	0.94 ± 0.17	0.94 ± 0.11	0.90 ± 0.14	0.81 ± 0.24	1.00 ± 0.00	0.91 ± 0.12	0.97 ± 0.08	1.00 ± 0.00	0.94 ± 0.17	0.97 ± 0.08
0.86	0.97 ± 0.08	1.00 ± 0.00	0.94 ± 0.17	0.97 ± 0.08	0.97 ± 0.08	1.00 ± 0.00	0.94 ± 0.17	0.97 ± 0.08	0.90 ± 0.14	0.81 ± 0.24	1.00 ± 0.00	0.91 ± 0.12	0.93 ± 0.13	0.94 ± 0.17	0.94 ± 0.17	0.94 ± 0.11
0.87	0.97 ± 0.08	1.00 ± 0.00	0.94 ± 0.17	0.97 ± 0.08	0.97 ± 0.08	1.00 ± 0.00	0.94 ± 0.17	0.97 ± 0.08	0.90 ± 0.14	0.81 ± 0.24	1.00 ± 0.00	0.91 ± 0.12	0.93 ± 0.13	0.94 ± 0.17	0.94 ± 0.17	0.94 ± 0.11
0.88	0.97 ± 0.08	1.00 ± 0.00	0.94 ± 0.17	0.97 ± 0.08	0.97 ± 0.08	1.00 ± 0.00	0.94 ± 0.17	0.97 ± 0.08	0.90 ± 0.14	0.81 ± 0.24	1.00 ± 0.00	0.91 ± 0.12	0.93 ± 0.13	0.94 ± 0.17	0.94 ± 0.17	0.94 ± 0.11
0.89	0.97 ± 0.08	1.00 ± 0.00	0.94 ± 0.17	0.97 ± 0.08	0.94 ± 0.11	1.00 ± 0.00	0.88 ± 0.22	0.94 ± 0.11	0.90 ± 0.14	0.81 ± 0.24	1.00 ± 0.00	0.91 ± 0.12	0.97 ± 0.08	1.00 ± 0.00	0.94 ± 0.17	0.97 ± 0.08
0.9	0.97 ± 0.08	1.00 ± 0.00	0.94 ± 0.17	0.97 ± 0.08	0.93 ± 0.13	0.94 ± 0.17	0.94 ± 0.17	0.94 ± 0.11	0.90 ± 0.14	0.81 ± 0.24	1.00 ± 0.00	0.91 ± 0.12	0.97 ± 0.08	1.00 ± 0.00	0.94 ± 0.17	0.97 ± 0.08
0.91	0.97 ± 0.08	1.00 ± 0.00	0.94 ± 0.17	0.97 ± 0.08	0.90 ± 0.14	0.81 ± 0.24	1.00 ± 0.00	0.91 ± 0.12	0.90 ± 0.14	0.81 ± 0.24	1.00 ± 0.00	0.91 ± 0.12	0.97 ± 0.08	1.00 ± 0.00	0.94 ± 0.17	0.97 ± 0.08
0.92	0.97 ± 0.08	1.00 ± 0.00	0.94 ± 0.17	0.97 ± 0.08	0.93 ± 0.13	0.94 ± 0.17	0.94 ± 0.17	0.94 ± 0.11	0.83 ± 0.18	0.69 ± 0.35	1.00 ± 0.00	0.84 ± 0.17	0.97 ± 0.08	1.00 ± 0.00	0.94 ± 0.17	0.97 ± 0.08
0.93	0.97 ± 0.08	1.00 ± 0.00	0.94 ± 0.17	0.97 ± 0.08	0.90 ± 0.14	0.94 ± 0.17	0.88 ± 0.22	0.91 ± 0.12	0.83 ± 0.18	0.69 ± 0.35	1.00 ± 0.00	0.84 ± 0.17	0.97 ± 0.08	1.00 ± 0.00	0.94 ± 0.17	0.97 ± 0.08
0.94	0.97 ± 0.08	1.00 ± 0.00	0.94 ± 0.17	0.97 ± 0.08	0.93 ± 0.13	0.94 ± 0.17	0.94 ± 0.17	0.94 ± 0.11	0.83 ± 0.18	0.69 ± 0.35	1.00 ± 0.00	0.84 ± 0.17	0.97 ± 0.08	1.00 ± 0.00	0.94 ± 0.17	0.97 ± 0.08
0.95	0.97 ± 0.08	1.00 ± 0.00	0.94 ± 0.17	0.97 ± 0.08	0.97 ± 0.08	1.00 ± 0.00	0.94 ± 0.17	0.97 ± 0.08	0.83 ± 0.18	0.69 ± 0.35	1.00 ± 0.00	0.84 ± 0.17	0.97 ± 0.08	1.00 ± 0.00	0.94 ± 0.17	0.97 ± 0.08
0.96	0.97 ± 0.08	1.00 ± 0.00	0.94 ± 0.17	0.97 ± 0.08	0.97 ± 0.08	1.00 ± 0.00	0.94 ± 0.17	0.97 ± 0.08	0.83 ± 0.18	0.69 ± 0.35	1.00 ± 0.00	0.84 ± 0.17	0.93 ± 0.13	0.94 ± 0.17	0.94 ± 0.17	0.94 ± 0.11
0.97	0.94 ± 0.11	0.94 ± 0.17	0.94 ± 0.17	0.94 ± 0.11	0.93 ± 0.13	0.94 ± 0.17	0.94 ± 0.17	0.94 ± 0.11	0.83 ± 0.18	0.69 ± 0.35	1.00 ± 0.00	0.84 ± 0.17	0.97 ± 0.08	1.00 ± 0.00	0.94 ± 0.17	0.97 ± 0.08
0.98	0.94 ± 0.11	0.94 ± 0.17	0.94 ± 0.17	0.94 ± 0.11	0.97 ± 0.08	1.00 ± 0.00	0.94 ± 0.17	0.97 ± 0.08	0.83 ± 0.18	0.69 ± 0.35	1.00 ± 0.00	0.84 ± 0.17	0.97 ± 0.08	1.00 ± 0.00	0.94 ± 0.17	0.97 ± 0.08
0.99	0.94 ± 0.11	0.94 ± 0.17	0.94 ± 0.17	0.94 ± 0.11	0.97 ± 0.08	1.00 ± 0.00	0.94 ± 0.17	0.97 ± 0.08	0.83 ± 0.18	0.69 ± 0.35	1.00 ± 0.00	0.84 ± 0.17	0.97 ± 0.08	1.00 ± 0.00	0.94 ± 0.17	0.97 ± 0.08
